# Traditional Chinese Medicine Text Similarity Calculation Model Based on the Bidirectional Temporal Siamese Network

**DOI:** 10.1155/2021/2337924

**Published:** 2021-11-29

**Authors:** Jigen Luo, Wangping Xiong, Jianqiang Du, Yingfeng Liu, Jianwen Li, Dingxing Hu

**Affiliations:** ^1^School of Computer, Jiangxi University of Chinese Medicine, Nanchang 330004, Jiangxi, China; ^2^Qihuang Academy, Jiangxi University of Chinese Medicine, Nanchang 330004, Jiangxi, China

## Abstract

The text similarity calculation plays a crucial role as the core work of artificial intelligence commercial applications such as traditional Chinese medicine (TCM) auxiliary diagnosis, intelligent question and answer, and prescription recommendation. However, TCM texts have problems such as short sentence expression, inaccurate word segmentation, strong semantic relevance, high feature dimension, and sparseness. This study comprehensively considers the temporal information of sentence context and proposes a TCM text similarity calculation model based on the bidirectional temporal Siamese network (BTSN). We used the enhanced representation through knowledge integration (ERNIE) pretrained language model to train character vectors instead of word vectors and solved the problem of inaccurate word segmentation in TCM. In the Siamese network, the traditional fully connected neural network was replaced by a deep bidirectional long short-term memory (BLSTM) to capture the contextual semantics of the current word information. The improved similarity BLSTM was used to map the sentence that is to be tested into two sets of low-dimensional numerical vectors. Then, we performed similarity calculation training. Experiments on the two datasets of financial and TCM show that the performance of the BTSN model in this study was better than that of other similarity calculation models. When the number of layers of the BLSTM reached 6 layers, the accuracy of the model was the highest. This verifies that the text similarity calculation model proposed in this study has high engineering value.

## 1. Introduction

The calculation of text similarity is a basic research in the field of natural language processing. It compares the similarity between two texts through a certain strategy to obtain a quantified similarity [1]. This technology is widely used in information retrieval [2], intelligent question and answer [3], and article plagiarism detection [4]. With the development of Chinese medicine, the government, hospitals, and universities are all carrying out the informatization, modernization, and industrialization of Chinese medicine. The electronic cases archived in hospitals have attracted more and more attention. The excavation of medical diagnosis information from the electronic cases is of great benefit to inheriting the experiences of Chinese medicine diagnosis and treatment. Text similarity calculation has important applications in the recommendation of Chinese medicine prescription, question point identification of intelligent question and answer models, and adjuvant therapy.

TCM text condenses the wisdom and experience of various Chinese medicine clinicians, but it requires refinement in expression. Therefore, the formed text has many characteristics, such as short sentence length, strong contextual relevance, and rich semantics [5]. Therefore, the results of some common basic natural language processing tasks, such as named entity recognition extraction, entity relationship extraction, and text similarity calculation, are poor. This is a great challenge faced by researchers in Chinese medicine. Using the traditional text similarity calculation model, we obtained highly similar sentence feature vectors for “can bone metastases of breast cancer be cured?” and “breast cancer skin metastasis can be cured.” However, the semantics of the two sentences are completely different. In order to solve the problem of low accuracy of TCM text similarity, we need to find a method to extract all the semantic information of sentences, reduce feature dimension and sparsity, and improve the accuracy of text similarity calculation. It is better applied to other tasks of natural language processing.

In this study, Section 2 reviewed the literatures on text similarity, Section 3 focused on the text similarity calculation model of TCM based on BTSN. In Section 4, the new method was tested and analyzed on two sets of data. Comparing with several existing algorithms, the effectiveness and feasibility of the new method were further verified. Finally, it was summarized and analyzed in Section 5.

## 2. Literature Review

At present, there are more and more methods to calculate text similarity, but most of the research on text similarity focuses on the public domain. Due to characteristics of short sentence length and strong semantic relevance, the research of TCM diagnostic text is slow. With the rapid development of natural language technology, scholars have proposed many methods to calculate the semantic similarity of text. The methods accepted by researchers are divided into the following three categories:

1. The methods based on large-scale domain knowledge or rules: the method based on knowledge rule matching [6] and the method based on syntactic analysis [7]. It is necessary to define grammatical rules in advance to build a large-scale dictionary, conceptual ontology, or knowledge base. Although it is efficient, it has a large investment in preliminary preparation. It is difficult to increase and update rules and knowledge base, which are dependent on the professional knowledge of linguists.
2. The method based on machine learning: the traditional vector space model (word bag model) [8], latent Dirichlet allocation (LDA) based topic model [9], BM25 algorithm for evaluating the correlation between text and keywords [10], FastText model [11], and so on. Typically, Yulianto et al. [12] used the Rabin–Karp algorithm for string matching and used the Jaro–Winkler algorithm to calculate the similarity of two short text sequences. The text similarity method based on traditional machine learning is more suitable for datasets with few samples. By establishing the quantitative relationship between text and feature vector, we can find the appropriate calculation method of vector and distance, so as to achieve the text similarity. However, these methods cannot fully express the semantic information of sentences in the feature vector part, and the feature dimension is high. These results show that accuracy of the model was insufficient.
3. The text similarity calculation method based on deep learning is the research direction in recent years [13]. This method trains distributed word vectors through the deep neural network. The feature vector dimension of the traditional bag-of-words model is high and sparse [14]. We can map words to low-dimensional space through distributed word vector. This not only sets word meaning vector value to words but also vectorizes the semantic differences between words to make them more distinguishable. Zeng et al. [15] proposed an emotional word embedding (ewe) model for subsequent tasks such as text classification and text similarity calculation.

Kusner et al. proposed the word move distance (WMD) text similarity calculation method based on deep learning [16]. Xie et al. [17] proposed a deep semantic structured model (DSSM) to calculate the vectorization of each document and then took the cosine value of the two text vectors as the similarity. Ruan et al. [18] calculated similarity by the embedding method. Li et al. [19] proposed a similarity calculation method based on the word meaning vector model. Li et al. [20] tried to combine the structural information and semantic information of sentences and simplified the processing of sentences into feature vectors by the Word2Vec model. Then, they inputted the syntactic structure of LTP to form the features of the sentence similarity calculation model. Later, scholars focused on the twin network, which was first proposed by Chopra et al. [21]. It is widely used in target tracking and face recognition. It is a neural network structure based on a group of networks with the same parameters. The twin network has the good modeling ability for texts with the same structure because of the symmetry from the same parameters. Subsequently, more and more scholars used the twin neural network to act on the text similarity tasks. They used the neural network shared by two weights to extract effective features through nonlinear mapping. Zhao et al. [22] pointed out that the use of asymmetric twin neural network for case correlation analysis is essentially to calculate the similarity of text cases.

The text similarity calculation in TCM is mainly used for the task of prescription recommendation. Li et al. [23] used the complex network to mine the core formula and calculated the similarity between the two formulas by the Jaccard similarity coefficient. Zhu et al. [24] explored the implicit relationship of prescription syndrome components by the LDA topic model. They transformed prescription component into two probability distributions of prescription syndrome type and syndrome component and used KL distance to calculate the similarity. The results showed that the TCM text similarity calculation model has made outstanding contributions.

In order to solve the problems of short sentences, inaccurate word segmentation, strong semantic correlation, high dimension of feature vector matrix, and sparse features of TCM text, we proposed a similarity calculation model of TCM text based on the bidirectional temporal Siamese network (BTSN). The pretrained language model ERNIE was used in this model to train the character vector instead of the word vector to solve the problem of inaccurate word segmentation in TCM. The BTSN structure was used to map the text pair to be tested into two low-dimensional vectors. The BLSTM was used to replace the traditional fully connected neural network to capture the context semantic information of the current word. It is helpful to extract more semantic information of sentences.

## 3. Bidirectional Temporal Siamese Network (BTSN)

**Figure 1:**
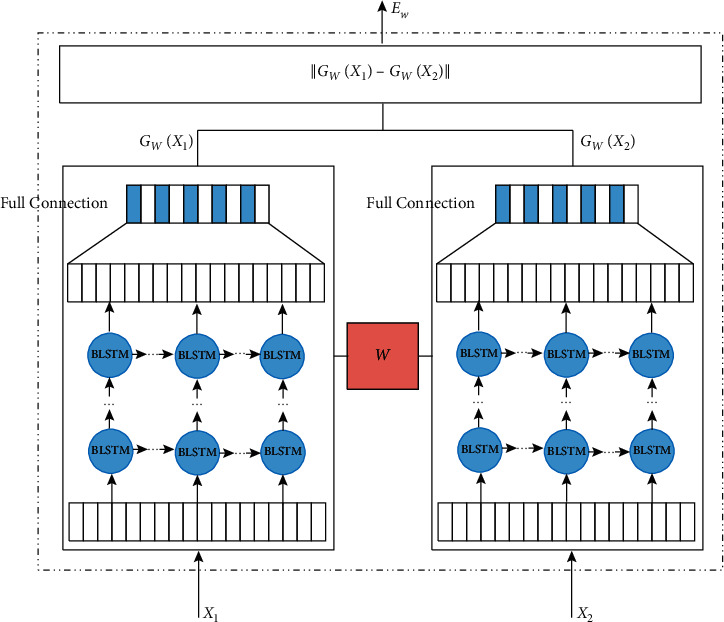
Text similarity calculation model based on BTSN.

The similarity calculation model of TCM text based on BTSN converts the similarity calculation into the classification task. When the similarity of the two texts to be tested is large, it is a positive class; otherwise, it is determined as a negative class. The model is divided into two parts. In the whole model structure, Siamese network framework is used to replace the traditional fully connected network in the weight-sharing network with BLSTM, so as to capture more text deep features and context semantic information. The model structure is shown in Figure 1.

The pseudocode of the algorithm of the TCM text similarity calculation model based on BTSN is as follows (Algorithm 1).

We will introduce the three basic parts of the BTSN model, which are character embedding, Siamese network, and LSTM network.

### 3.1. Character Embedding

The neural network model cannot directly process text data, so it needs to convert text type data into a numerical vector. There are two ways of text vectorization: one hot and distributed representation. Since distributed vector representation can reduce the dimension of vectors and effectively represent the association between semantics, this study adopts the distributed representation method for text vectorization. Before entering the model, the text was embedded at the character level. In this study, the text was converted into the character vector through the pretrained language model, called the ERNIE model [25]. We took the multiinformation entities in the knowledge map as external knowledge to improve the language representation and enhance the representation of the pretrained language model of bidirectional encoder representations from transformers (BERT) [26].

The input sentence is *S*, the set of words contained in this sentence is *W*(*w*_1_, *w*_2_, *w*_3_,…, *w*_*m*_), and *m* is the sentence length. The word vector of the *t* word is *w*_*t*_^*∗*^ ∈ *R*^*d*^, where the dimension of the feature vector in the above equation is *d*. The input text is

(1) $$\begin{array}{c} S=\left[w_{1}{}^{*},w_{2}{}^{*},\ldots ,w_{m}{}^{*}\right]\in R^{T\times d}. \end{array}$$

In Figure 1, the input text *X*_1_ and *X*_2_ are similar text sentence pairs that will be tested. After character embedding, the input text entered the bidirectional LSTM neural network model with shared weights. The overall framework was the Siamese network, and the internal fully connected neural network was replaced by bidirectional LSTM. The LSTM network is improvement of the recurrent neural network (RNN). Its main purpose is to solve the problem of gradient disappearance or gradient explosion and make full use of context information to mine more hidden features.

### 3.2. Siamese Network

**Figure 2:**
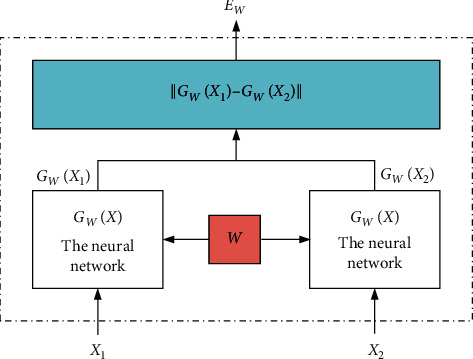
The framework of the Siamese network.

The Siamese network is a connected neural network with weight sharing. It was first published in 2005 for judging facial similarity. The Siamese neural network has two input terminals. In specific tasks, two data are input into the neural network with weight sharing. Through nonlinear mapping to the new space, we calculated loss function in the new space to measure the similarity between the two input data. The framework of the Siamese network is shown in Figure 2.

In Figure 2, *X*_1_ and *X*_2_ are the two input similarity samples that will be tested. Through the traditional neural network shared by the two weights, the feature can be transformed into another feature space through nonlinear mapping, and two output vectors can be obtained to evaluate whether the two input samples are similar, *G*_*W*_(*X*_1_) and *G*_*W*_(*X*_2_). In the Siamese network, the energy function *E*_*W*_(*X*_1_, *X*_2_) is used to compare the outputs of two weight-sharing networks. The energy function is

(2) $$\begin{array}{c} E_{W}\left(X_{1},X_{2}\right)=\left\|G_{W}\left(X_{1}\right)-G_{W}\left(X_{2}\right)\right\|. \end{array}$$

The loss function is related to the input and parameters, and the loss function of the Siamese network is

(3) $$\begin{array}{c} L\left(W,{\left(Y,X_{1},X_{2}\right)}^{i}\right)=\left(1-Y\right)L_{G}\left(E_{W}{\left(X_{1},X_{2}\right)}^{i}\right)+YL_{I}\left(E_{W}{\left(X_{1},X_{2}\right)}^{i}\right),\\ K\left(W\right)=\sum_{i=1}^{p}L\left(W,{\left(Y,X_{1},X_{2}\right)}^{i}\right), \end{array}$$

where (*Y*, *X*_1_, *X*_2_)^*i*^ represents the *i*^th^ sample, which is composed of a pair of test similarity text and its label. *L*_*G*_ is the loss function of text pairs of the same category, *L*_*I*_ is the function of text pairs of different categories, and *p* is the total number of training samples. By designing different loss function expressions, we can reduce the energy of the same category pair and increase the energy of the different category pair.

### 3.3. LSTM Network

**Figure 3:**
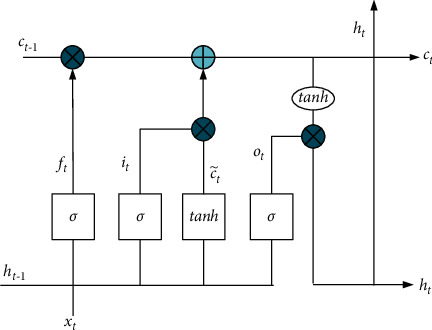
LSTM unit structure.

We replaced the traditional neural network by LSTM in Siamese because of its strong memory ability. In addition, it can accurately grasp the overall semantic information of sentences and has strong feature expression ability. The difference from the RNN is the neuron. The neural unit of the LSTM neural network is joined with gate structure, and the neural unit of the LSTM neural network is shown in Figure 3.

There are three gates in the structure diagram of LSTM unit: input gate, output gate, and forget gate. Due to its special structure, LSTM can solve the problem of long-distance dependence to some extent.

At time *t*, the component update status of each LSTM unit is

(4) $$\begin{array}{c} f_{t}=\sigma \left(W_{f}\cdot \left[h_{t-1},x_{t}\right]+b_{f}\right),\\ i_{t}=\sigma \left(W_{f}\cdot \left[h_{t-1},x_{t}\right]+b_{i}\right),\\ \tilde{c_{t}}=\tanh\left(W_{c}\cdot \left[h_{t-1},x_{t}\right]+b_{c}\right),\\ c_{t}=f_{t}\circ c_{t-1}+i_{t}\circ \tilde{c_{t}},\\ o_{t}=\sigma \left(W_{o}\cdot \left[h_{t-1},x_{t}\right]+b_{o}\right),\\ h_{t}=o_{t}\circ \;\tanh\left(c_{t}\right), \end{array}$$

where *σ* represents the activation function sigmoid, ∘ is the element multiplication, *x*_*t*_ is the input vector of LSTM at the time *t*, *h*_*t*_ represents the implied state, *W*_*f*_, *W*_*i*_, *W*_*c*_, and *W*_*o*_ represent the weight matrix of forgetting gate, input gate, memory unit, and output gate, respectively, *b*_*f*_, *b*_*i*_, *b*_*c*_, *b*_*o*_ represent the bias of forgetting gate, input gate, memory unit, and output gate, respectively, *f*_*t*_*i*_*t*_, *c*_*t*_, and *o*_*t*_ represent the forgetting gate, input gate, memory unit state, and output gate, respectively.

In order to make full use of context information of sentences, we mined more implied features to effectively solve the problem of feature extraction. In this study, we used BLSTM to replace the Siamese ordinary neural network structure. This method is composed of two opposite direction LSTM network. The model structure is shown in Figure 1. $\overset{⟶}{h_{t}}$ is the LSTM, prior to the output of the neural network in *t* time, and $\overset{\leftarrow }{h_{t}}$ is after the LSTM output at time *t*. Therefore, the output for the first time *t* back to joining together is $h_{t}=\left[\overset{⟶}{h_{t}},\overset{\leftarrow }{h_{t}}\right]$.

## 4. Experiment and Analysis

### 4.1. Experiment

In order to verify the feasibility and effectiveness of the improved text similarity calculation model proposed in this study, we compared experiments on two sets of data of TCM and finance through the TCM text similarity calculation model based on BTSN. The example of TCM text similarity dataset is given in Table 1.

The experimental evaluation index in this study is the accuracy on the training set and the test set. In order to give the formula of the accuracy more intuitively, the required confusion matrix is given in Table 2.

Based on the confusion matrix in Table 2, the accuracy is

(5) $$\begin{array}{c} \text{Accuracy}=\frac{TP+TN}{TP+FN+FP+TN}. \end{array}$$

In order to comprehensively verify the advantages of the improved text similarity calculation model proposed in this study, we set three comparative experiments. Experiment 1 is a comparative experiment of training character vector and word vector with various pretrained language models as the input of the network. The purpose is to verify which language model was selected in the embedding layer and whether character level or word level vector has been selected as the network input; the second experiment is the experimental comparison between BTSN and other algorithms. This verifies that BTSN is more scientific than other text similarity algorithms; experiment 3 is an in-depth network depth experiment of BTSN to find the optimal number of network layers.

### 4.2. The Comparison of Character and Word Input Vectors of Different Pretrained Models

In experiment 1, three pretrained language models, Word2Ver, BERT, and ERNIE, were selected to train character vector and word vector as the input of the BTSN model. Word2Vec training word vector was the input of BTSN (WW-BTSN), Word2Vec was used to train the character vector as the input of BTSN (WC-BTSN), BERT training word vector was the input of BTSN (BW-BTSN), BERT training character vector was the input of BTSN (BC-BTSN), ERNIE training word vector was the input of BTSN (EW-BTSN), and ERNIE training character vector was the input of BTSN (EC-BTSN). In experiment 1, the input vector dimension was uniformly set to 300 dimensions, the epoch was uniformly set to 20 times, and the network depth was 2 layers. The model results are given in Table 3.

Comparing the word and character vectors trained by the pretrained model on the two sets of data in Table 3, we found that the effect of the ERNIE model was significantly better than that of the other pretraining models. ERNIE added knowledge information from entities on the basis of the original semantic information of the BERT model. However, the Word2Vec model is a static word embedding model. It is highly limited in expressing semantic information.

Comparing character input vector with word input vector, we found that character vector as the input of the text similarity calculation model is better than word vector input. In the financial training dataset, the accuracy of EC-BTSN was 0.8328. It was 1.15% greater than that of the EW-BTSN model. In the financial test set, the accuracy of the EC- BTSN model was 0.8311. It was 2% greater than that of the EW-BTSN model, reached 0.8102.

In the training set of TCM, the accuracy of the EC-BTSN model was 0.7967. It was 2.36% greater than that of EW-BTSN, reached 0.7731. In the TCM test set, the accuracy of the EC-BTSN model was 0.7827. It was 2.19% greater than that of EW-BTSN. In addition, the effect of using character vector as the input of the neural network model was remarkable.

The results of above six groups of comparative experiments show that selecting character vector as model input can improve the accuracy of evaluation indicators to varying degrees. This is because the mainstream word segmentation tools have serious inaccurate word segmentation problems in TCM. Due to a large number of terminology in TCM, it is impossible to achieve accurate word segmentation through stuttering word segmentation and other tools. For example, in TCM terminology, “yin-yang deficiency syndrome” will be divided into yin-yang, two, and deficiency syndrome through the stuttering word segmentation tool. In TCM, this phrase should be taken as a whole and cannot be separated.

### 4.3. The Comparison of Different Similarity Calculation Models

**Figure 4:**
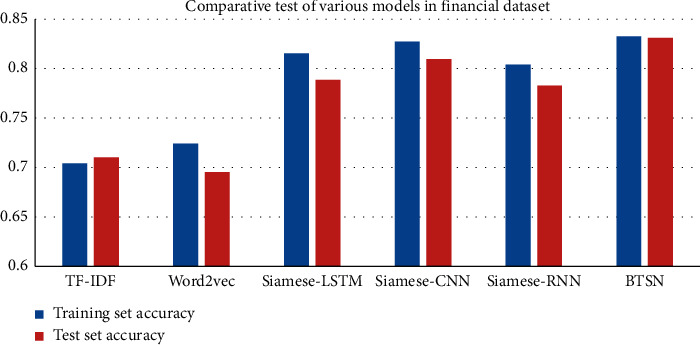
Histogram of accuracy of each model in the financial dataset.

**Figure 5:**
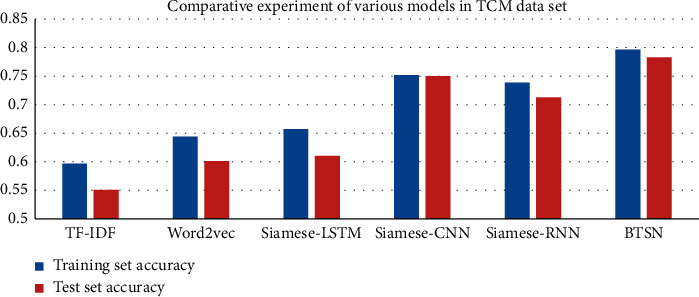
Histogram of accuracy of each model in the TCM dataset.

In experiment 2, we set up six groups of comparison models, represented by term frequency-inverse document frequency (TF-IDF) feature, to calculate the cosine distance of the similarity text [27]. Word2Vec was used to represent the character level vector. After weighted average of all characters in the sentence, the similarity was determined by the cosine distances [28]. The Siamese-LSTM model is for text vector extraction using straightaway LSTM. We replaced the fully connected network in Siamese with the CNN and paid more attention to local information of sentences in the Siamese-CNN model; The sentence semantic information and extract the Siamese-RNN model of sentence overall features were captured by RNN. The forward and backward semantic information of sentences was captured by the bidirectional LSTM neural network. The overall features of sentences were extracted through the BTSN model; the comparison results of six groups are given in Table 4 and Figures 4 and 5.

The experimental results of the BTSN text similarity calculation model and other five models are given in Table 4 and Figures 4 and 5. The improved algorithm BTSN proposed in this study has better results on two sets of data.

The TF-IDF model constructs feature vectors based on the sentence word frequency statistics, without the semantic information between words. It has the disadvantages of high feature dimensions and sparseness. Therefore, the result of similarity calculation is not good enough. The accuracy in the financial training dataset was 0.7042 and that in the test set was 0.7102. The accuracies in the training set and test set of TCM were 0.5967 and 0.5510, respectively. There is still a lot of room for improvement.

In order to solve the shortcomings of the TF-IDF model, we mapped high-dimensional features to heterogeneous space in the Word2vec model. This further reduced feature dimensions and ensured strong semantic relationship between characters. The accuracies of this method in the financial training set and test set were 0.7242 and 0.6954. Compared with the TF-IDF model, the accuracy in the training set was improved by 2%, and the accuracy in the test set was reduced by 1.148%. However, the experimental effect on the TCM dataset was significant. The accuracies of the training set and test set were 0.6441 and 0.6013, which was nearly 5% greater than that of the TF-IDF model. This indicates that considering the semantic information of sentences has more advantages over the text similarity calculation model.

The text similarity calculation model based on Word2vec weighted the average vectors of all characters in the sentence after character embedding, without the nonlinear relationship between character vectors. In order to solve this problem, this study proposed a text similarity calculation model based on Siamese-LSTM. In Table 4, the effect of the Siamese-LSTM model on two sets of datasets has been greatly improved. The accuracies of the Siamese-LSTM model on the financial training set and test set were 0.8156 and 0.7886, respectively. They were 9% greater than that of the Word2Vec model. The accuracy of the Siamese-LSTM model on the training set and test set of TCM was 0.6574 and 0.6103, respectively. They were 1% greater than that of the Word2Vec model.

Considering the local information of sentences extracted by the CNN, this study proposed a text similarity calculation model based on the Siamese-CNN. In Table 4, on the financial data training set, the accuracy of the Siamese-CNN was 0.8273, which is 1% greater than that of the Siamese-LSTM model. In contrast, on the financial test set, the accuracy of the Siamese-CNN was 0.8097, which was also greater than that of Siamese-LSTM. The accuracies of the Siamese-CNN for the training set and test set of TCM were 0.7517 and 0.7502, respectively. They were 9.43% and 13.99% greater than that of the Siamese-LSTM model. Local features sentences play an important role in the text similarity calculation model.

We set the Siamese-RNN model to verify the advantages of LSTM over the RNN model. We found that there are many advantages of LSTM from the two groups of test data of the Siamese-LSTM and Siamese-RNN. The accuracies of financial data and TCM data have been improved to a certain extent. Furthermore, the experiments also verified that the gate structure provided by LSTM can alleviate the problem of gradient disappearance or gradient explosion. In addition, we can mine more hidden features through the full use of context information.

Finally, based on the Siamese-LSTM model, a layer of reverse LSTM neuron structure was added to form a Siamese network with bidirectional LSTM structure, that is, BTSN. The purpose is to capture more context information of sentences based on the nonlinear transformation. In Table 4, the experimental results show that context information of sentences is very important for feature extraction. The accuracies in the financial training set and test set were 0.8328 and 0.8311, respectively. They were greater than that of other algorithms. However, the experimental effect on the TCM dataset was more significant. The accuracies of the improved model in its training set and test set were 0.7967 and 0.7827, respectively. They were 4.5% and 3.25% greater than that of the Siamese-CNN model. The improved BTSN model proposed in this study has the better experimental effect on text similarity calculation. The bidirectional LSTM network can extract useful features. It has a better and significant effect on the text of short sentences in TCM.

### 4.4. The Comparison of Different Network Layers

**Figure 6:**
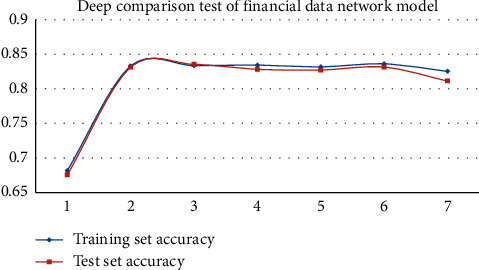
The experimental diagram of network layers of the financial dataset.

**Figure 7:**
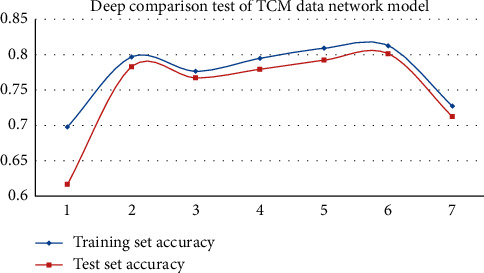
The experimental diagram of network layers of the TCM dataset.

In experiment 3, we set different depths of the BLSTM networks to find the optimal number of network layers. In this experiment, the layers of BTSN models were from layer 1 to layer 7. The experimental results of each layer are given in Table 5 and Figures 6 and 7.

According to the graphs of the network layer experiment, the accuracy of the BTSN text similarity calculation model on the two sets of datasets was increased, with the increasing depth of the BLSTM. When the depth of the network was 6 layers, the accuracies of the training set and the test set of the financial dataset were 0.8361 and 0.8314, respectively. The accuracies of training set and test set of TCM data were 0.8125 and 0.8014, respectively. After that, with the continuous increase of network depth, the effect of the model was decreased, and the training time was increased. Therefore, when the network depth of the BTSN text similarity calculation model is 6 layers in this experiment, the experimental effect is the best.

Finally, this study proposes a TCM text similarity calculation model based on BTSN, with certain advantages. It can overcome the problems of short sentences, inaccurate word segmentation, strong semantic correlation, high dimension of feature vector matrix, and sparse features of TCM diagnosis text. We comprehensively considered the semantic time series information of the sentence context and improved the accuracy of the model. The experimental effect is more scientific.

## 5. Conclusions

Due to the sentence characteristics of TCM text, the higher and sparse dimension of the vector matrix in constructing sentence features, we comprehensively considered the time sequence information of sentence context and proposed a TCM text similarity calculation model based on the bidirectional temporal Siamese network (BTSN). The model uses the ERNIE pretrained language model to train character vectors instead of traditional word vectors to solve the problem of inaccurate word segmentation in TCM; the traditional fully connected neural network was replaced by the deep BLSTM network in the Siamese to capture the context timing information of the current word. This is conducive to extract more semantic information of sentences. Through experiments on two sets of datasets of TCM and financial, it is proved that the BTSN model has certain advantages over other models. When the depth of the BLSTM network is 6 layers, the accuracy of the two sets of data can reach the best. This can be well applied to the calculation of text similarity. For the feature extraction of TCM short text, we will further increase the effective features related to sentence expression through external knowledge base or domain knowledge map.

## Figures and Tables

**Algorithm 1:**
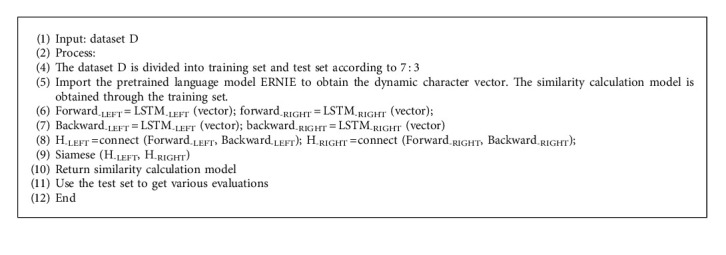
Bidirectional temporal Siamese network (BTSN).

**Table 1:** Samples of the TCM data.

Sentence 1	Sentence 2	Similarity label
Hypertension, coronary heart disease how to treat	Can hypertension give birth to children?	0
Can people with diabetes drink yoghurt	Diabetes drink yoghurt line	1
What are the symptoms of breast cancer terminal?	What are the symptoms of early breast cancer?	0
Is breast hyperplasia and breast cancer related?	What is the incidence of breast cancer?	0

**Table 2:** Confusion matrix.

	The prediction is a positive class	The prediction is negative
The actual is class	Is actually positive and predicted to be TP	It is actually positive and it is predicted to be negative (FN)
The actual negative class	The actual value is negative and the prediction is FP	It is actually negative, and it is predicted to be negative (TN)

**Table 3:** The comparison of experimental results of word vector.

	Financial data		TCM data	
	Training set accuracy	Test set accuracy	Training set accuracy	Test set accuracy
WW-BTSN	0.7054	0.6764	0.6283	0.5986
WC-BTSN	0.8170	0.7861	0.6661	0.6503
BW-BTSN	0.7634	0.7276	0.7023	0.6891
BC-BTSN	0.8192	0.7943	0.7483	0.7239
EW-BTSN	0.8213	0.8102	0.7731	0.7608
EC-BTSN	**0.8328**	**0.8311**	**0.7967**	**0.7827**

Bold values indicate the best record in the comparative test.

**Table 4:** The comparison of different similarity calculation models.

Datasets	Financial data		TCM data	
Models	Training set accuracy	Test set accuracy	Training set accuracy	Test set accuracy
TF-IDF	0.7042	0.7102	0.5967	0.5510
Word2vec	0.7242	0.6954	0.6441	0.6013
Siamese-LSTM	0.8156	0.7886	0.6574	0.6103
Siamese-CNN	0.8273	0.8097	0.7517	0.7502
Siamese-RNN	0.8039	0.7830	0.7383	0.7129
BTSN	**0.8328**	**0.8311**	**0.7967**	**0.7827**

Bold values indicate the best record in the comparative test.

**Table 5:** The number of layers in the BTSN model network.

Network layers	Financial data		TCM data	
	Training set accuracy	Test set accuracy	Training set accuracy	Test set accuracy
1 network layer	0.6819	0.6756	0.6979	0.6166
2 network layers	0.8328	0.8311	0.7967	0.7827
3 network layers	0.8335	0.8353	0.7766	0.7673
4 network layers	0.8342	0.8282	0.7948	0.7794
5 network layers	0.8318	0.8272	0.8091	0.7922
6 network layers	**0.8361**	**0.8314**	**0.8125**	**0.8014**
7 network layers	0.8253	0.8113	0.7274	0.7124

Bold values indicate the best record in the comparative test.

## Data Availability

The datasets generated and/or analyzed during the current study are available from the corresponding author upon request.

## Abbreviations

TCM: Traditional Chinese medicine
BERT: Bidirectional encoder representations from transformers
ERNIE: Enhanced representation through knowledge integration
LSTM: Long short-term memory
CNN: Convolutional neural network
RNN: Recurrent neural network.
